# Metacognitions in patients with chronic obstructive pulmonary disease: a psychometric study of the metacognitions questionnaire-30

**DOI:** 10.3389/fpsyg.2023.1265102

**Published:** 2023-10-20

**Authors:** Toril Dammen, Costas Papageorgiou, Jonas Christoffer Lindstrøm, Gunnar Einvik

**Affiliations:** ^1^Institute of Clinical Medicine, Faculty of Medicine, University of Oslo, Oslo, Norway; ^2^Department of Research and Innovation, Division of Mental Health and Addiction, Oslo University Hospital, Oslo, Norway; ^3^Department of Psychology, University of Oslo, Oslo, Norway; ^4^Department of Method Development and Analytics, Norwegian Institute of Public Health, Oslo, Norway; ^5^Health Services Research Unit (HØKH), Akershus University Hospital, Lørenskog, Norway; ^6^Department of Pulmonary Medicine, Akershus University Hospital, Lørenskog, Norway

**Keywords:** chronic obstructive lung disease, metacognitions, anxiety, depression, psychometrics, metacognitive therapy

## Abstract

The metacognitions questionnaire-30 (MCQ-30) was developed for the assessment of metacognitive beliefs and processes that are central components of the metacognitive model of emotional disorders. Anxiety and depression commonly occur in patients with chronic obstructive pulmonary disease (COPD). Testing such a model for anxiety and depression in patients with COPD is warranted. However, the psychometric properties of the MCQ-30 in COPD patients are unknown. Therefore, in this study we aimed to examine these properties in COPD patients. The MCQ-30 was administered to 203 COPD patients referred to a rehabilitation unit in respiratory medicine. Confirmatory factor analysis (CFA) was used to test the five-factor as well as the bi-factor models of MCQ-30. Exploratory factor analyses were also performed. Both models did not meet the criteria for an acceptable fit on Comparative Fit Index (CFI) of 0.810 and 0.858 vs. criterion of ≥0.9, but the Root Mean Square Error of Approximation (RMSEA) criterion ≤0.08 was acceptable for both models with RMSEA = 0.074 and 0.066, respectively. The factors were mostly moderately correlated (0.41–0.58) with acceptable reliability coefficients (0.73–0.87). The exploratory factor analysis identified three of the five factors originally described in the five-factor model of the MCQ-30. These data show that the factor structure of the MCQ-30 appears to differ from that of the original instrument in COPD patients and further studies are needed to confirm its validity and reliability in this patient group.

## Introduction

Chronic obstructive pulmonary disease (COPD) is characterized by persistent, largely irreversible airway obstruction and is one of the top three causes of death worldwide (Halpin et al., 2019). Clinical symptoms of anxiety and depression are significant and modifiable conditions in COPD patients and more commonly occur in COPD patients than in those with other chronic diseases such as cancer, diabetes, or heart disease (Marsh and Guck, 2015). Up to 55% of COPD patients suffer from clinical anxiety (Hynninen et al., 2005) and 40% is considered a benchmark prevalence for depression in stable COPD patients (Yohannes et al., 2000) whereas up to 86% of those with acute exacerbation may present with depression (Lecheler et al., 2017). The presence of anxiety and depression has important implications for COPD patients including: increased mortality risk (Einvik et al., 2015), higher re-admission rates, poor health behaviors (i.e., higher levels of smoking and lower levels of physical exercise; Paine et al., 2019), poor quality of life (Ma et al., 2020), poor physical health status, higher risk of exacerbations (Xu et al., 2008) as well as frequent use of acute healthcare services (Farver-Vestergaard et al., 2018).

Together these important consequences highlight the need to develop and implement effective interventions to reduce psychological distress in this patient group. Recent reviews of the effectiveness of psychological interventions for anxiety and depression in COPD patients point to small effect sizes (Smith et al., 2014). Cognitive behavioral therapy (CBT) has produced small to medium effect sizes (Farver-Vestergaard et al., 2018; Ma et al., 2020) prompting some authors to call for more well-designed studies (e.g., Smith et al., 2014; Farver-Vestergaard et al., 2018).

Given the lack of highly effective interventions for depression and anxiety in these patients, and the fact that anxiety and depression commonly co-occur, there is a need to develop more effective transdiagnostic treatments. One such approach that targets both anxiety and depression and thus may be effective for COPD patients with clinical anxiety and depression is metacognitive therapy (MCT; Wells, 2009). The study of metacognition in cancer (Cook et al., 2014, 2015; Fisher et al., 2016) and heart disease (Faija et al., 2020) has recently improved the psychological treatment of anxiety and depression in these conditions (e.g., Fisher et al., 2016; Wells et al., 2021). Therefore, we believe that the study of metacognition in COPD may enhance our understanding of these processes in anxiety and depression and improve current psychotherapeutic approaches.

Metacognition can be defined as “the knowledge of knowledge” or “cognition about cognitive phenomena” (Flavell, 1979). Nelson (1996) referred to metacognition as a function that acts as a monitor and controller over our cognitive processes. Whereas Wells developed a metacognitive model based on the S-ref model, Lysaker et al. (2021) developed an integrated model of metacognitions in patients with psychosis that may be interpreted as an umbrella concept (Fekete et al., 2022).

Metacognitive interventions include those with therapeutic elements targeting metacognitions. They may be based on different theoretical models and the effectiveness has been reviewed for a broader range of such therapies (Philipp et al., 2018). More specifically, MCT (Wells, 2009) is grounded on the metacognitive model that is based on the Self-Regulatory Executive Function (S-REF) model of emotional disorders (Wells and Matthews, 1996). It emphasizes biased metacognition (i.e., thinking about thinking) as a central mechanism responsible for psychological distress and disorders. Furthermore, metacognitive beliefs (e.g., “worrying will control my symptoms,” “worrying will damage my health”) give rise to a preservative negative processing style referred to as the “cognitive attentional syndrome” (CAS; Wells, 2009). The CAS comprises perseverative thinking (i.e., worry and rumination), unhelpful attentional strategies, such as threat monitoring, and unhelpful coping strategies (e.g., excessive rest to become less depressed, avoidance of activities). Applying this to COPD, potential factors that may activate the CAS include worrying about future worsening of symptoms, ruminating on loss of function, or fear about disease progression. In those who become anxious or depressed, sustained rumination or worry occurs because these processes are driven by specific metacognitions. The goal of MCT is to modify metacognitive beliefs and processes that contribute to the maintenance of psychological distress. Meta-analytic and systematic reviews have concluded that MCT is an effective intervention for symptoms of anxiety and depression (Normann and Morina, 2018). Furthermore, it has been argued that MCT may be a more appropriate and effective psychological intervention for those with anxiety and depression in patients with physical illness because, unlike other treatments such as traditional CBT, it does not focus on realistic versus unrealistic thought content but instead the processes responsible for perpetuating excessive and unhelpful mental activity (Wells, 2009). MCT addresses preservative thinking in terms of rumination and worry in order to alleviate distress. And MCT would rather question the benefits of worry about future COPD exacerbation than evaluate whether the worry is unrealistic or not.

In order to test the metacognitive model and therapy among COPD patients, a valid and reliable measure of metacognitions is required. Metacognition, a trans-diagnostic construct based on different theoretical models, can be measured through numerous tools, as recently reviewed by Martiadis et al. (2023). However, the Metacognitions Questionnaire-30 (MCQ-30) has been the most widely used self-report measure of metacognitions (Wells and Cartwright-Hatton, 2004). The MCQ-30 comprises the following five subscales: Positive beliefs about worry; negative beliefs about the danger and uncontrollability of worry; beliefs about the need to control thoughts; cognitive self-consciousness; and cognitive confidence. Initial evaluations of the MCQ-30 consistently identified a five-factor model with acceptable fit as well as good internal consistency, acceptable test–retest reliability, and convergent validity with measures of anxiety, depression, and worry (Wells and Cartwright-Hatton, 2004). To date, the psychometric properties of the MCQ-30 have been studied in samples in the United Kingdom (Spada et al., 2008), Turkey, Korea (Cho et al., 2012), Serbia (Marković et al., 2019), in clinical samples (Martin et al., 2014; Grøtte et al., 2016; Bright et al., 2018), and in patients with somatic illness (Cook et al., 2014; Fisher et al., 2016). In all of these studies, the five-factor structure was identified and correlated significantly with depression, anxiety, and worry.

In more recent studies, attempts have been made to use the metacognitive model to study anxiety and depression in patients with chronic physical illnesses such as cancer (Cook et al., 2014), Parkinson’s disease (Brown and Fernie, 2015), epilepsy (Fisher et al., 2016), and cardiac disease (Faija et al., 2020). Whereas three of these studies identified the five-factor model and found excellent reliability estimates for all of the subscales (i.e., Cook et al., 2014; Brown and Fernie, 2015; Fisher et al., 2016), Faija et al. (2020) did not replicate this model and recommended cautious continuous use of the MCQ-30 among cardiac disease patients. However, the psychometric properties of the MCQ-30 in COPD patients have not been investigated, in COPD patients and, eventually, the metacognitive-based psychotherapy interventions in this population. This is necessary in order to test the metacognitive model in COPD patients.

Although previous studies across various samples have suggested a five-factor structure with significant correlations between the subscales (Wells and Cartwright-Hatton, 2004; Spada et al., 2008; Ramos-Cejudo et al., 2013; Cook et al., 2014), two studies conducted in a non-clinical sample and in cardiac patients provided preliminary support for a bi-factor model of the MCQ-30 (see Fergus and Bardeen, 2019, for further details). The bi-factor model consisted of the same five factors as previously identified with the addition of a general factor contributing to all the individual items. Since this is the first psychometric study of MCQ-30 in COPD patients, and a recent study in cardiac patients has proposed a better fit for the bi-factor model (Faija et al., 2020), we wanted to investigate both the original five-factor and the recently explored bi-factor model.

Furthermore, consistent with key hypotheses of the S-REF model, systematic reviews and meta-analyses provide support of positive relationships between depression, anxiety, and metacognitions (Rochat et al., 2017; Normann and Morina, 2018). Studies examining these relationships in somatic disease are scarce. However, positive relationships between metacognitions, anxiety, and depression have been identified in patients with cancer (Cook et al., 2015), Parkinson’s disease (Allott et al., 2005), epilepsy (Fisher et al., 2016), and cardiac illness (Anderson et al., 2019; Faija et al., 2020). However, these relationships are not known in COPD patients.

## Aims

With this background, this study aims to explore for the first time the psychometric properties of the MCQ-30 in COPD patients. The primary aim was to explore the established and original five-factor structure of the MCQ-30 or the more recently explored bi-factor structure in this population and to investigate the internal consistency of its subscales. A second aim was to estimate the associations between the specific MCQ-30 subscales and anxiety and depression. Finally, we aimed to examine which specific subscales of the MCT-30 are better predictors of anxiety and depression symptoms in patients with COPD. Consistent with the results of previous studies across various clinical samples (e.g., Spada et al., 2008; Fisher et al., 2016; Grøtte et al., 2016; Marković et al., 2019; Capobianco et al., 2020; Zhang et al., 2020), we hypothesized that the Negative beliefs about worry subscale would be the main predictor of both anxiety and depression in COPD patients. This would also be consistent with key predictions of the metacognitive model.

## Materials and methods

### Ethics statement

This research was approved by the Norwegian Research Ethics Committee (reference: 2018/149) and the local data protection officer. All participants signed their written informed consent form prior to data collection. There are no conflicts of interest to be declared.

### Participants and procedure

Consecutive patients attending a 4-week inpatient pulmonary rehabilitation program at the LHL Hospital Gardermoen in South-Eastern Norway over a 6-month period from June 2018 were approached for participation. Inclusion criteria were: a diagnosis of COPD, aged over 18 years, no cognitive impairments, and sufficient understanding of the Norwegian language. During the first days of their hospital stay, the patients received oral and written information about the study. Patients consenting to participate received and completed the questionnaires within the first week of their stay. Study coordinators employed at the LHL hospital Gardermoen collected the questionnaires.

In total, 249 patients were assessed for eligibility, 34 declined to participate, and 10 did not meet inclusion criteria due to having a respiratory disease other than COPD. Two participants who had initially consented to participate were later excluded as the diagnosis of COPD was revised. Hence, the total study population consisted of 203 patients. The sample and procedures have been described in more detail elsewhere (Garratt et al., 2022).

### Measures

The Metacognitions Questionnaire-30 (MCQ-30; Wells and Cartwright-Hatton, 2004) is a self-report questionnaire that assesses metacognitive beliefs across five subscales: (1) Positive beliefs about worry (e.g., “Worrying helps me to avoid problems in the future”); (2) Negative beliefs about uncontrollability and danger of worry (e.g., “My worrying is dangerous for me,” “When I start worrying I cannot stop”); (3) Cognitive confidence (e.g., “I have little confidence in my memory for words and names”); (4) Need to control thoughts (e.g., “I should be in control of my thoughts all of the time”); and (5) Cognitive self-consciousness (e.g., “I think a lot about my thoughts”). For each subscale, items are scored on a four-point scale (1 = do not agree, 2 = agree slightly, 3 = agree moderately, and 4 = agree very much), yielding total scores ranging from 6 to 24. More positive and negative beliefs about worry, reduced confidence in memory, greater belief in the need to control thoughts, and an increased tendency toward self-focused attention are indicated by higher scores. The MCQ-30 has been shown to have high internal consistency and good convergent and predictive validity (Wells and Cartwright-Hatton, 2004; Spada et al., 2008). Adequate psychometric properties have been reported in obsessive-compulsive disorder patients for the Norwegian version of the questionnaire that was used in the present study (Grøtte et al., 2016).

The Hospital Anxiety and Depression scale (HADS; Zigmond and Snaith, 1983) was used to assess anxiety and depression. The HADS is a well-established self-report measure of emotional distress specifically developed for use in physically ill populations. Fourteen items are scored on a four-point scale yielding two subscale scores ranging from 0 to 21 with high scores indicating greater anxiety or depressive symptoms. The HADS yields two seven-item subscale scores for symptoms of anxiety (HADS-A) and depression (HADS-D). The HADS has been validated for use in COPD patients (Nowak et al., 2014; Phan et al., 2016) and is one of the most widely employed measures of anxiety and depression symptoms in this population. It is recommended for use in pulmonary rehabilitation in Europe (Nyberg et al., 2021). The Norwegian version of this scale has been reported to have good reliability across studies and in patients with physical illness (Leiknes and Siqveland, 2016).

### Statistical analysis

Descriptive statistics include means and standard deviations for the MCQ-30 and the HADS for the total sample. Confirmatory factor analyses (CFA) were used to fit factor models of both the original five-factor structure and a bi-factor model of the MCQ-30. The original five-factor model has five correlated factors while the bi-factor model has one general factor that all items load on, in addition to the five original subfactors that in this case are uncorrelated.

To investigate whether the data could support alternative factor structures, we also conducted an exploratory factor analysis (EFA). The number of factors was chosen using the eigenvalue-greater-than-1 rule, inspection of the Scree Plot, and oblimin rotation was used.

The adequacy of the models was principally assessed by two statistical indices that are least sensitive to sample size and parameter estimates (Hu and Bentler, 1998): the Comparative Fit Index (CFI) and the Root Mean Square Error of Approximation (RMSEA) along with its 90% confidence interval. A CFI of 0.90 or above is commonly taken to indicate an acceptable fit (Kline, 2016), and previous studies of the MCQ-30 have all used this criterion value (e.g., Wells and Cartwright-Hatton, 2004; Grøtte et al., 2016; Faija et al., 2020). However, Hu and Bentler (1999) have argued for a level of 0.95. In order to compare our results with those of previous studies, we used 0.90. RMSEA less than 0.08 indicates an acceptable fit, with a limit of 0.1 or less as the upper 90% confidence limit.

We also estimated secondary indices: the Goodness of Fit Index (GFI), with values closer to 1 indicating good fit; the Parsimony Goodness of Fit Index (PGFI), for which values above 0.5 indicate good fit (Hu and Bentler, 1999); and because we aimed to establish comparisons with previous studies reporting the Tucker-Lewis Fit Index (TLI), for which 0.90 represents a good fit (Garver and Mentzer, 1999), we also included this index. Chi-square statistic is also reported. We did not base our goodness-of-fit decisions on this criterion because it is very sensitive to sample size and to high correlations between factors, hence having been described as inappropriate for detecting well-fitting models. For all other indexes 0.90 was considered adequate and 0.95 good (Bentler and Bonett, 1980; Hu and Bentler, 1999).

We first began by fitting the pre-specified five-factor and bi-factor models. Then, subsequently we used exploratory factor analysis to examine if an alternative solution emerged that provided a better model fit. Inter-correlations between the original factors were investigated with Pearson’s test and Cronbach’s alpha was used as a measure of internal consistency of the HADS and MCQ-30 subscales.

**Figure 1:**
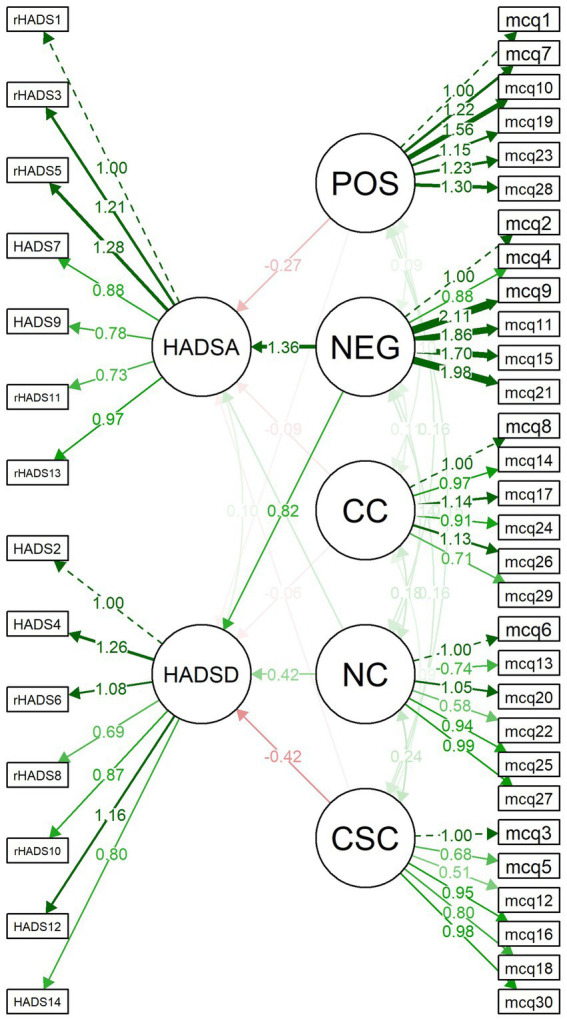
Structural equation modeling of the relationship between anxiety (HADSA: Hospital Anxiety and Depression Scale, anxiety subscale) and depression (HADSD: Hospital Anxiety Depression Scale, depression subscale) and Metacognitions Questionnaire-30 (MCQ-30) subscales (POS: positive beliefs about worry; NEG: negative beliefs about uncontrollability and danger; CC: cognitive confidence; NC: negative beliefs about the need to control thoughts; CSC: cognitive self-consciousness).

Structural Equations Modeling (SEM) was used for regression analysis of MCQ-30 subfactors against HADS depression and anxiety subscales. Each MCQ-30 and HADS subscale was treated as latent variables, and all five MCQ-30 subscales were used as predictors of the two HADS subfactors (see Figure 1). Age and gender were also included as predictors of depression and anxiety.

Fifty-two individual responses were missing (0.6%) for MCQ-30 and HADS. No item had more than 2% missing, except MCQ item 28 with 9% missing responses; missing values were handled with listwise deletion, and FIML estimate for the SEM analyses.

All analyses were conducted in R (v 4.0.2). The CFA and SEM analyses were computed using the Lavaan package (Rosseel, 2012), and the EFA was performed using the psych and GPA rotation packages.

## Results

### Sample demographics and descriptive statistics

The sample consisted of 203 participants. The mean age (SD) was 65.2 (9.0), 49% were female, and 54% were receiving disability benefits. Table 1 shows demographics and the means and standard deviations for the HADS and the MCQ-30 scores.

**Table 1:** Demographic characteristics, HADS and MCQ scores (*N* = 203).

	Mean, (SD)	*n* (%)
Age		
	65.2 (9.0)	
Gender		
Females		100 (49.3)
Males		103 (50.7)
Employment status		
Receiving disability benefits		110 (54.2)
Retired		58 (28.7)
Sick leave		19 (9.4)
In work		29 (14.4)
HADS		
Anxiety	7.1 (4.4)	
Depression	6.1 (3.8)	
MCQ-30		
Positive beliefs	9.0 (3.1)	
Negative beliefs	12.4 (4.2)	
Cognitive confidence	11.7 (4.2)	
Need for control	10.8 (3.6)	
Cognitive self-consciousness	12.0 (3.5)	
Total	55.9 (13.7)	

HADS, Hospital and anxiety depression scale; MCQ-30, Metacognition questionnaire-30; and SD, Standard deviation.

### HADS and MCQ-30 subscale correlations and internal consistencies

Table 2 shows that all of the inter-correlations between the MCQ-30 subscales were all significant and ranged from 0.19 to 0.58. The Cronbach alpha values ranged from 0.73 to 0.87 for the MCQ-30 subscales. All of the correlations between the MCQ-30 subscales and the HADS-D were significant, and ranged from 0.15 to 0.43, and for the HADS-A ranged from 0.27 to 0.60 (Table 2).

**Table 2:** Correlations between HADS and MCQ-30 subscales (five-factor original subscales) and internal consistencies.

	HADSA	HADSD	POS	NEG	CC	NC	CSC	Alpha
HADSA	1	0.66^**^	0.37^**^	0.60^**^	0.27^**^	0.38^**^	0.36^**^	0.88
HADSD	0.66^**^	1	0.37^**^	0.43^**^	0.29^**^	0.38^**^	0.15^**^	0.80
POS	0.37^**^	0.37^**^	1	0.44^**^	0.43^**^	0.58^**^	0.47^**^	0.79
NEG	0.60^**^	0.43^**^	0.44^**^	1	0.37^**^	0.51^**^	0.53^**^	0.79
CC	0.27^**^	0.29^**^	0.43^**^	0.37^**^	1	0.41^**^	0.19^**^	0.87
NC	0.38^**^	0.38^**^	0.58^**^	0.51^**^	0.41^**^	1	0.54^**^	0.73
CSC	0.36^**^	0.15^**^	0.47^**^	0.53^**^	0.19^**^	0.54^**^	1	0.73

HADS, Hospital anxiety and depression scale; HADSA, Hospital anxiety and depression scale—anxiety subscale; HADSD, Hospital anxiety and depression scale—depression subscale; MCQ-30, Metacognitions questionnaire-30; POS, Positive beliefs about worry; NEG, Negative beliefs about worry; CC, Cognitive confidence; NC, Need to control thoughts; and CSC, Cognitive self-consciousness.

** *p* < 0.001.

### Factor structure

#### Confirmatory factor analysis

Goodness-of-fit statistics for the models are presented in Table 3. The original five-factor model showed acceptable fit according to the RMSEA criteria. However, the CFI at 0.810 was below the threshold for acceptability. The secondary fit criteria showed mixed results with acceptable fit for the GFI and PGFI but not for the TLI. The bi-factor model showed a CFI closer to acceptable fit and acceptable fit according to the RMSEA criterion. Fit was acceptable according to the GFI and PGFI but not to the TLI. The fit indices indicated a better fit for the bi-factor model.

**Table 3:** Goodness of fit indices for the five-factor and bi-factor models.

Models	Fit measures								
	*X* ^2^	df	*p*	CFI	RMSEA	90% CI	TLI	GFI	PGFI
Five-factor	836.135	395	<0.001	0.810	0.074	(0.067–0.081)	0.791	0.907	0.724
Bi-factor	704.459	375	<0.001	0.858	0.066	(0.058–0.073)	0.836	0.933	0.707

*X*^2^, Chi-square; df, Degrees of freedom; CFI, Comparative fit index; RMSEA, Root mean square error of approximation; CI, Confidence interval; TLI, Tucker-Lewis Index; GFI, Goodness of fit index; and PGFI, Parsimony goodness of fit index.

#### Exploratory factor analyses

We performed exploratory factor analyses using the eigenvalue-greater-than-1 rule, scree plot, and oblimin rotation. These analyses identified a five-factor structure. The factor analyses are shown in Table 4.

**Table 4:** MCQ-30 scale structure and items and factor loadings for each MCQ item on factors.

	Factors				
MCQ-30 subscales and items	1	2	3	4	5
*Positive beliefs*					
1. Worrying helps me to avoid problems in the future	0.26	0.03	−0.20	0.12	0.22
7. I need to worry in order to remain organized	0.28	−0.02	−0.20	0.04	0.37
10. Worrying helps me to get things sorted out in my mind	**0.55**	0.01	−0.38	0.11	0.14
19. Worrying helps me cope	0.15	−0.06	−0.12	0.02	**0.56**
23. Worrying helps me to solve problems	0.20	0.18	−0.34	0.15	0.23
28. I need to worry in order to work well	0.09	0.16	−0.20	0.04	**0.52**
*Negative beliefs*					
2. My worrying is dangerous for me	0.21	−0.06	**0.57**	0.07	0.13
4. I could make myself sick with worrying	0.12	0.03	**0.56**	0.25	0.01
9. My worrying thoughts persist, No matter how I try to stop them	**0.72**	0.08	0.05	0.01	0.03
11. I cannot ignore my worrying thoughts	**0.62**	0.12	0.08	0.12	−0.06
15. My worrying could make me go mad	**0.58**	0.14	0.36	−0.03	−0.04
21. When I start worrying I cannot stop	**0.85**	0.00	0.02	−0.08	0.05
*Cognitive confidence*					
8. I have little confidence in my memory for Words and names	0.16	**0.65**	−0.14	0.07	−0.04
14. My memory can mislead me at times	0.11	**0.69**	0.03	0.15	−0.12
17. I have a poor memory	−0.05	**0.92**	−0.01	−0.03	−0.04
24. I have little confidence in my memory for places	−0.02	**0.62**	0.00	−0.07	0.14
26. I do not trust my memory	−0.06	**0.82**	0.05	−0.06	0.10
29. I have little confidence in my memory for actions	0.11	**0.44**	−0.05	−0.03	0.26
*Need to control thoughts*					
6. If I did not control a worrying thought, and then It happened, it would be my fault	0.10	0.15	0.14	0.06	**0.42**
13. I should be in control of my thoughts all of the time	0.01	0.03	0.06	**0.48**	0.15
20. Not being able to control my thoughts is a sign of weakness	0.18	0.08	0.09	−0.06	**0.57**
22. I will be punished for not controlling certain thoughts	0.24	0.08	0.06	−0.08	**0.42**
25. It is bad to think certain thoughts	−0.04	0.07	0.22	0.05	**0.55**
27. If I could not control my thoughts, I would not be able to function	−0.15	0.05	0.02	0.32	**0.55**
*Cognitive Self Consciousness*					
3. I think a lot about my thoughts	**0.66**	−0.08	−0.01	0.08	0.09
5. I am aware of the way my mind works when I am thinking through a problem	−0.01	0.02	0.23	**0.53**	0.04
12. I monitor my thoughts	−0.03	−0.05	−0.08	**0.67**	−0.15
16. I am constantly aware of my thinking	0.11	0.05	0.06	**0.51**	0.17
18. I pay close attention to the way my mind works	0.02	−0.04	0.06	**0.61**	0.10
30. I constantly examine my thoughts	0.46	−0.04	−0.09	0.29	0.13

Loadings >0.4 are bold.

As shown in Table 4, for one of the original factors (CC) all items loaded >0.4 on factor 2. However, one item (MCQ-29) cross-loaded on two factors (factor 2 and factor 5). For the NC subscale, five of the original items loaded on one factor (factor 5), while the MCQ-13 item “I should be in control of my thoughts all the time” loaded highest on factor 4. Furthermore, MCQ-27 item cross-loaded on two factors (factor 4 and 5). For the CSC subscale, four out of six items loaded on factor 4, whereas one item MCQ-3 (i.e., “I think a lot about my thoughts”) loaded highest on factor 1 and MCQ-30 item “I constantly examine my thoughts” cross-loaded on factor 1 and 4. Regarding the positive beliefs about worry subscale, MCQ items 1, 7, and 23 did not load >0.4 on any factor. The MCQ-10 item (“Worrying helps me get things sorted out in my mind”) loaded most strongly on factor 1 whereas items MCQ-19 and MCQ-28 loaded on factor 5. Four items from the original negative beliefs scale loaded 0.4 or above on factor 1 whereas two items, MCQ-2 and MCQ-4, loaded strongly on factor 3.

#### MCQ-30 structural equation modeling analyses

The results of the analysis showed that only the negative beliefs subscale was significantly associated with HADS-anxiety after we controlled for age and gender (*p* < 0.001). Inclusion of the MCQ-30 subscales accounted for 61.6% of the variance in HADS-Anxiety, with also age being an independent significant predictor (*p* = 0.020). The MCQ-30 domain-specific subscales were significant and accounted for 38.4% of the variance in predicting HADS-depression, with the negative belief subscale (*p* = 0.002), need to control thoughts (*p* = 0.031) and cognitive self-consciousness (*p* = 0.04) being significantly associated with HADS-depression (Figure 1).

## Discussion

This is the first study with the principal aim to confirm and explore the factor structure of MCQ-30 in patients with COPD. In summary, the results only partially supported the original five-factor model as most fit indices did not meet criteria. However, in line with previous studies, all of the original MCQ-30 subscales were positively and significantly correlated with each other. Moreover, the Cronbach alphas were acceptable ≥0.7. Consistent with the conclusions reached by Faija et al. (2020), the results of our study suggest that the original MCQ-30 five-factor model may not be ideal at the present time for use with COPD patients until additional psychometric studies of this scale in this clinical population are systematically conducted and replicated.

The bi-factor model results demonstrated a better fit to the data and suggest that it contains more information beyond the five-factor model, as also found by Faija et al. (2020). In addition to one previous non-clinical study, our study represents the second clinical investigation in patients with somatic disease to have found a bi-factor model accounting for the factors of the MCQ-30. Wells and colleagues concluded that “It is also computationally much more complex to derive scores from the bi-factor solution and their interpretation is not as simple” (Faija et al., 2020, p. 7). In line with Faija et al. (2020), we agree that for practical purposes it would be useful to continue to use the original MCQ-30 subscales in COPD patients because these subscales demonstrated good internal consistency and correlated positively and significantly with each other and with anxiety and depression.

Consistent with previous studies across various samples (Wells and Cartwright-Hatton, 2004; Spada et al., 2008; Yilmaz et al., 2008; Cho et al., 2012; Cook et al., 2014; Martin et al., 2014; Fisher et al., 2016; Grøtte et al., 2016), we found that the MCQ-30 and its subscales had adequate reliability in COPD patients.

We also examined predictors of symptoms of anxiety and depression. As hypothesized, the negative beliefs about uncontrollability and danger subscale was the strongest independent predictor of both types of symptoms and was the only subscale that significantly predicted higher anxiety scores. This result provides further evidence for the metacognitive model (Wells, 2009). This model emphasizes the importance of negative metacognitive beliefs contributing to the development of chronic anxiety. Additional metacognitive predictors of depression were beliefs about the need to control thoughts and cognitive self-consciousness. The negative metacognitive beliefs subscale has been identified as the strongest predictor of depression, although findings about other metacognitive predictors are inconsistent (Wells and Papageorgiou, 1998; Yilmaz et al., 2008; Cho et al., 2012). The MCQ-30 subscale assessing beliefs about the need to control thoughts has previously been identified as an independent predictor of depression (Spada et al., 2008) in line with our results. These finding emphasizes the importance of thought control in the development and maintenance of depressive symptoms.

To sum up, consistent with the results of previous studies, we found positive relationships between symptoms of anxiety, depression, and metacognitions. Furthermore, the MCQ-30 negative beliefs subscale was the strongest predictor of anxiety and depression and the only subscale that significantly and positively correlated with anxiety. The clinical implication of this finding is that MCT targeting negative beliefs may contribute to reductions in symptoms of anxiety and depression among patients with COPD. In addition, the subscales “need to control thoughts” and “cognitive self-consciousness” contributed significantly to depression with a negative relationship for the cognitive self-consciousness subscale. This suggests that lower cognitive self-consciousness was associated with stronger symptoms of depression that is in line with what was reported by Faija et al. (2020) in cardiac patients.

Furthermore, our results only partially supported the original five-factor model. In line with our hypothesis, we identified a five-factor structure, but the EFA only partly identified the original five-factor structure as we found four scales, but the positive beliefs about worry subscale was not identified as the items belonging to this original scale loaded on different factors. Interestingly, items belonging to the negative beliefs subscale loaded on different factors. This is consistent with the results of a recent study suggesting that the negative beliefs factor might comprise two subfactors, one with MCQ2 and 4 (Nordahl et al., 2022). We may speculate that these items may represent a subfactor of danger whereas the other items are more likely to represent uncontrollability. The clinical implications of two factors, particularly in patients with physical illness that might tend to worry about danger, remains to be further explored.

### Strengths and limitations

This study has two main strengths: a large sample size >200 patients and missing data were very small. Our study also has a number of limitations: Only self-report measures were used; the test–retest reliability of the MCQ-30 was not examined, the bi-factor model did not meet the more stringent criterion of Hu and Bentler (1999) for goodness of fit and the sample was without distinctions in COPD stages that may have influenced results. Furthermore, the small sample size and the specific patient group derived from only one hospital rehabilitation center may limit the generalizability of the findings.

### Conclusion

The results of the present study indicated that the original factor structure of the MCQ-30 may not be generalizable to COPD patients at this point in time. However, consistent with recent studies, the results showed that the bi-factor structure of this instrument demonstrated a slightly better fit to the data and warrants further systematic evaluations. Importantly, and in line with metacognitive theory, the results of this study showed that a number of specific metacognitive beliefs, especially those associated with uncontrollability and danger, predicted symptoms of anxiety and depression in individuals with COPD. Future studies are needed to further investigate the relationships between COPD, distress and metacognitive beliefs. Moreover, future evaluations of metacognitive therapy should be considered for anxiety and depression related distress in COPD patients.

## Data availability statement

According to Norwegian legislation, the Norwegian Data Protection Authority, and the Committee of Ethics, we are not allowed to share original study data publicly. However, the essential generated data are available from the corresponding author on reasonable request.

## Ethics statement

The studies involving humans were approved by Norwegian Research Ethics Committee. The studies were conducted in accordance with the local legislation and institutional requirements. The participants provided their written informed consent to participate in this study.

## Author contributions

TD: Conceptualization, Methodology, Resources, Writing – original draft. CP: Conceptualization, Methodology, Resources, Writing – review & editing. JL: Formal analysis, Investigation, Methodology, Software, Supervision, Writing – review & editing. GE: Conceptualization, Data curation, Funding acquisition, Investigation, Project administration, Resources, Writing – review & editing.
